# Ichthyosis with confetti: clinics, molecular genetics and management

**DOI:** 10.1186/s13023-015-0336-4

**Published:** 2015-09-17

**Authors:** Liliana Guerra, Andrea Diociaiuti, May El Hachem, Daniele Castiglia, Giovanna Zambruno

**Affiliations:** Laboratory of Molecular and Cell Biology, Istituto Dermopatico dell’Immacolata-IRCCS, Rome, Italy; Dermatology Unit, Bambino Gesù Children’s Hospital-IRCCS, Rome, Italy

**Keywords:** Ichthyosis with confetti, Ichthyosis variegata, Congenital reticular ichthyosiform erythroderma, Mammillae hypoplasia, Ear hypoplasia, Keratin 10, Keratin 1, Revertant skin, Loss of heterozygosity

## Abstract

Ichthyosis with confetti (IWC) is an autosomal dominant congenital ichthyosis also known as ichthyosis variegata or congenital reticular ichthyosiform erythroderma. It manifests at birth with generalized ichthyosiform erythroderma or with a collodion baby picture. The erythrodermic and ichthyotic phenotype persists during life and its severity may modify. However, the hallmark of the disease is the appearance, in childhood or later in life, of healthy skin confetti-like spots, which increase in number and size with time. IWC is a very rare genodermatosis, with a prevalence <1/1,000,000 and only 40 cases reported worldwide. The most important associated clinical features include ear deformities, mammillae hypoplasia, palmoplantar keratoderma, hypertrichosis and ectropion. IWC is due to dominant negative mutations in the *KRT10* and *KRT1* genes, encoding for keratins 10 and keratin 1, respectively. In this context, healthy skin confetti-like spots represent “repaired” skin due to independent events of reversion of keratin gene mutations via mitotic recombination. In most cases, IWC clinical suspicion is delayed until the detection of white skin spots. Clinical features, which may represent hint to the diagnosis of IWC even before appearance of confetti-like spots, include ear and mammillae hypoplasia, the progressive development of hypertrichosis and, in some patients, of adherent verrucous plaques of hyperkeratosis. Altogether the histopathological finding of keratinocyte vacuolization and the nuclear staining for keratin 10 and keratin 1 by immunofluorescence are pathognomonic. Nevertheless, mutational analysis of *KRT10* or *KRT1* genes is at present the gold standard to confirm the diagnosis. IWC has to be differentiated mainly from congenital ichthyosiform erythroderma. Differential diagnosis also includes syndromic ichthyoses, in particular Netherton syndrome, and the keratinopathic ichthyoses. Most of reported IWC cases are sporadic, but familial cases with autosomal dominant mode of inheritance have been also described. Therefore, knowledge of the mutation is the only way to properly counsel the couples. No specific and satisfactory therapy is currently available for IWC. Like for other congenital ichthyoses, topical treatments (mainly emollients and keratolytics) are symptomatic and offer only temporary relief. Among systemic treatments, retinoids, in particular acitretin, improve disease symptoms in most patients. Although at present there is no curative therapy for ichthyoses, treatments have improved considerably over the years and the best therapy for each patient is always the result of both physician and patient efforts.

**Electronic supplementary material:**

The online version of this article (doi:10.1186/s13023-015-0336-4) contains supplementary material, which is available to authorized users.

## Review

### Disease name/synonyms

Ichthyosis with confetti

Congenital reticular ichthyosiform erythroderma

Ichthyosis variegata

Orphanumber: ORPHA281190

OMIM: 609165

### Definition

Ichthyosis with confetti (IWC) [1] is an autosomal dominant congenital ichthyosis also known as ichthyosis variegata [2] or congenital reticular ichthyosiform erythroderma (CRIE) [3], the latter being the disease name recommended in the clinical-genetic classification of inherited ichthyoses, developed by the First Ichthyosis Consensus Conference in 2009 [4]. According to the current classification, IWC belongs to non-syndromic ichthyoses [4]. IWC manifests at birth with generalized ichthyosiform erythroderma [1, 3, 5–16] or with a collodion baby picture [17–19]. The hallmark of this form of ichthyosis is the appearance, in childhood or later in life, of confetti-like spots of pale and normal-appearing skin, which increase in number and size with time [1, 3, 5–19].

## Methods

A literature search was performed on PubMed from 1984, when IWC was first described, to April 30, 2015. In addition, the Orphanet database [20] was looked at. The following search terms were used: “ichthyosis with confetti”, “congenital reticular ichthyosiform erythroderma”, “ichthyosis variegata”, “MAUIE syndrome”. We then checked the bibliography of each article to identify additional references. Altogether, 17 reports containing a total of 40 IWC cases were identified.

### Epidemiology

IWC is a very rare genodermatosis, with a prevalence <1/1,000,000 [20]. To our knowledge, only 40 IWC cases have been reported (Table 1) [1, 3, 5–19]. The disease may be underdiagnosed as 9 cases have been described between 1984 and 2010 [1, 3, 5, 7, 8, 10–12], while ten cases have been collected by Choate et al. who deciphered the causative gene in 2010 [13] and 21 additional cases have been reported since then [15–17, 19].

**Table 1:** Clinical findings in 40 reported cases of ichthyosis with confetti

Case (Ref)	Age at first report, ys	Sex	CB/CIE at birth	HSS/age at detection, ys	S/HK	EM	MH	PPK	DAH	EE	Additional findings	Molecular analysis
^a^1 [1]	14	M	−/+	+/8	+/NR	NR	NR	NR	NR	NR	SS	NP
2 [3]	57	F	−/+	+/NR	+/+	NR	^f^+	+	NR	NR	HES, LNP, UI	NP
^b^3 [7]	17	M	−/+	+/since birth	+/NR	+	NR	-	-	+	AU, E, NMSC	NP
^b^4 [8]	30	F	−/+	+/NR	+/NR	+	NR	-	-	+	AU, NMSC	NP
5 [10]	8	F	−/+	+/years after	+/NR	NR	NR	+	+	NR	NR	NP
6 [11]	8	F	−/+	+/6	+/NR	NR	NR	+	+	+	DHL, P	NP
7 [12]	32	M	−/+	+/10	+/+	NR	^f^+	+	NR	NR	P	NP
^c^8 [17]	3	F	+/+	+/2.5	+/+	+	^c^+	+	+	+	GA, GH, JCF, ^c^LL/LNP, P, PR, SI, SS/LW, ^c^UI	*KRT10*: c.1383_1414del32 (exon 7), de novo
^c^9 [17]	6	M	+/+	+/5	+/+	+	^c^+	+	+	+	GA, JCF, ^c^LNP, P, ^c^SI, SS/LW, ^c^UI	*KRT10*: c.1374-1G > C (intron 6), de novo
10–29 [13, 15, 18]	18, 42, ^g^11, NR:17	^g^1F, 4 F 5 M NR:10	^g^+/+ NR:19	^g^+/8 NR:19	^g^+/+ NR:19	^g^ + NR:19	NR	^g^ + NR:19	NR	NR	^h^GA:8, ^g^JCF, ^h^NMSC:3, ^h^PH:4, ^g^UI	*KRT10*:
												c.1374-2A > G (intron 6), de novo
												c.1374-2delA (intron 6), de novo
												c.1374-1G > A (intron 6), de novo
												c.1373 + 1G > A (intron 6)
												c.1369G > T (exon 6), de novo
												c.1450insC (exon 7), de novo
												c.1560delCG (exon 7)
												NR: 13 *KRT10* mutations^h^
30 [19]	Child	F	+/+	+/7	+/NR	+	+	+	+	+	LL/LNP, P, REE, SI, SS/LW, UI	*KRT10*: c.1374-1G > A (intron 6), de novo
31 [19]	Young adult	M	+/+	+/12-13	+/NR	+	+	-	+	+	HHSS, LL/LNP, N, REE, S, SI, SS/LW	*KRT10*: c.1374-1G > C (intron 6), de novo
32 [1, 6, 9, 19]	12	F	−/+	+/10	+/+	+	+	+	+	+	DFL, HES/HHSS, JCF, LL/LNP, P, S, SS/LW, UI	*KRT10*: c.1506_1507delAA, (exon 7), de novo
33 [5, 14, 19]	5	F	−/+	+/8	+/NR	+	+	+	+	+	DFL, HHSS, JCF, LL/LNP, N, P, REE, S, SS/LW, UI	*KRT10*: c.1546_1551delinsT (exon 7), de novo
34 [19]	Child	F	+/+	+/7	+/NR	+	+	+	+	-	SS/LW	*KRT10*: c.1557_1558delCG (exon 7), de novo
35 [19]	Young adult	F	+/+	+/12-14	+/NR	NI	+	+	+	-	DFL, HHSS, LL/LNP, SS/LW, UI	*KRT10*: c.1573_1574dupA (exon 7), de novo
^d^36 [19]	Young adult	F	NR	NR	NR	NR	NR	NR	NR	NR	NR	*KRT10*: c.1573_1574dupA (exon 7), de novo
37 [16]	35	M	−/+	+/22	NR	NR	NR	+	NR	NR	NR	*KRT1*: c.1886insG (exon 9), de novo
^e^38–40 [16]	3 to 9	M	−/+	-	NR	NR	NR	+	NR	NR	NR	*KRT1*: c.1886insG (exon 9)

Most reported clinical findings: *CB/CIE* colloidon baby/congenital ichthyosiform erythroderma; *HSS* healthy skin spots; *S/HK* scaling/hyperkeratosis; *EM* ear malformation; *MH* mammillae hypoplasia; *PPK* palmoplantar keratoderma; *DAH* dorsal acral hypertrichosis; *EE* eyelid ectropion; + present; − absent

Additional findings: *AU* alopecia universalis; *DFL* decreased finger length (relative to palm). *DHL* diffuse hair loss; *E* eclabion; *GA* gait abnormality due to joint contractions of the limbs; *GH* generalized hypertrichosis; *HES* hyperpigmentation on erythrokeratotic skin; *HHSS* hyperpigmentation in healthy skin spots; *JCF* joint contractions of the fingers; *LL/LNP* large lunulae/ long nail plates; *N* nystagmus; *NMSC* nonmelanoma skin cancer; *P* pruritus. *PH* peripheral hyperreflexia; *PR* psychomotor retardation; *REE* reduced eyebrows and eyelashes; *S* strabismus; *SI* scalp involvement; *SS/LW* short stature/ low weight (relative to age); *UI* unguis inflexus; *NR* not reported. *NP* not performed

^a^Camenzind et al. [1] reported a second IWC patient (patient 32 in Table 1) who was further described by Brusasco et al. [6, 9] and Spoerri et al. [19]

^b^Elbaum et al. [7] and Hendrix et al. [8] reported two patients with a disease classified as MAUIE syndrome that shared almost all clinical findings with IWC

^c^Additional clinical data of patients first described by Diociaiuti et al. [17]

^d^Monozygotic twin sister of patient 35, with a very similar phenotype

^e^Affected offsprings of patient 37

^f^MH is visible on published pictures of Marghescu et al. [3] and Krunic et al. [12]

^g^a 11 years old female was clinically characterized by Long [18]

^h^symptoms reported by Choate et al. [15] in a IWC cohort of 20 patients all carrying *KRT10* mutations

### Clinical description

**Fig. 1:**
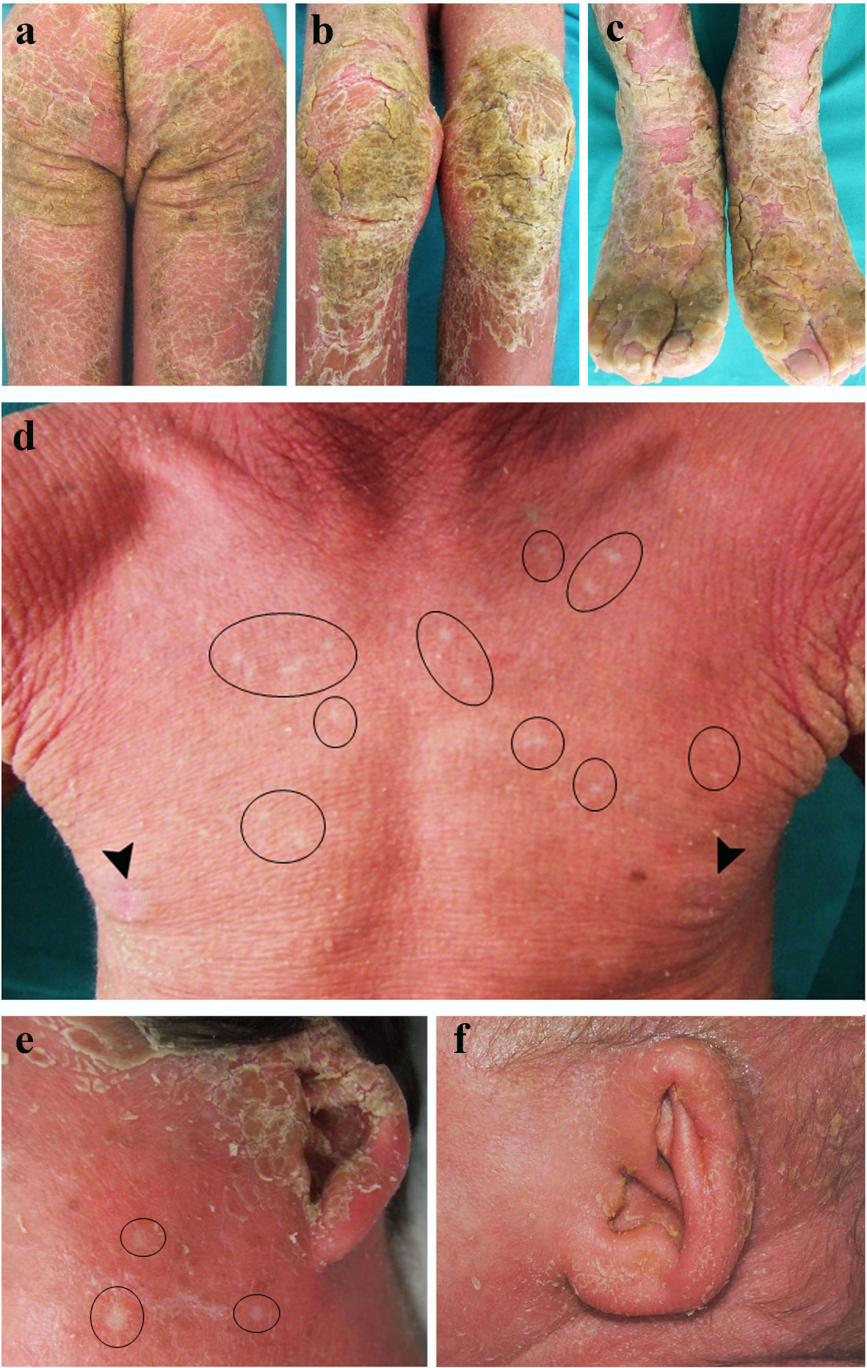
Major clinical features of ichthyosis with confetti. Severe ichthyosiform erythroderma: massive verrucous hyperkeratosis on the buttocks (**a**), knees (**b**) and feet (**c**) in the absence of retinoid therapy. Confetti-like spots of healthy skin are visible on the trunk and cheek (**d**, **e**: *black rings*) of the same patient. Note the presence of mammillae hypoplasia (**d**, *arrowheads*). Ear malformations: hypoplasia of the ear helix (**e**, **f**), and lobule (**e**). Clinical pictures are from a 8-year-old male (**a**-**e**) and a 1-year-old female (**f**)

IWC manifests at birth as a non-bullous generalized ichthyosiform erythroderma [1, 3, 10–12, 16, 19] or as a collodion baby [17–19]. The collodion membrane is typically shed within the first days of life [17–19]. The erythrodermic and ichthyotic phenotype (Fig. 1a–d) persists during life, even if its severity may modify [19]. In addition, the extent and type of scaling and hyperkeratosis vary significantly, ranging from white fine scales [11, 12], highly reminiscent of congenital ichthyosiform erythroderma, to verrucous hyperkeratotic adherent plaques more evident on the limbs [17] (Fig. 1a–c). Of note, with time patients develop confetti-like spots of healthy skin (Fig. 1d–e) on the erythrodermic background. These are the hallmark of the disease and frequently suggest the correct diagnosis. Spots start appearing during childhood, gradually increase in number to hundreds [13] and enlarge from 2 to 10 mm up to 4 cm in diameter [1, 11, 13, 14, 17]. Sometimes, they are recognized only after hyperkeratosis shedding due to retinoid therapy [1, 3, 5, 11, 12, 17]. The spots are regularly present on the trunk [6, 17, 18] and may spread to limbs and the rest of the body. Healthy spots seem to follow a distribution gradient across the skin, being largest and more numerous on the neck, décolleté and scapula regions, fewer and smaller on arms and legs, and rare on the face [19]. Moreover, they have been described either as depressed [1, 11] or slightly elevated [6] with respect to the surrounding skin. Due to the presence of many, at instance confluent, confetti-like spots, the adjacent ichthyotic skin may assume a reticulate pattern [3, 6, 12]. Of note, a later age at first appearance of confetti-like spots (22 years), a smaller size (maximum 4 mm) and a predominant involvement of flexures characterize the recently described IWC-II subtype [16].

**Fig. 2:**
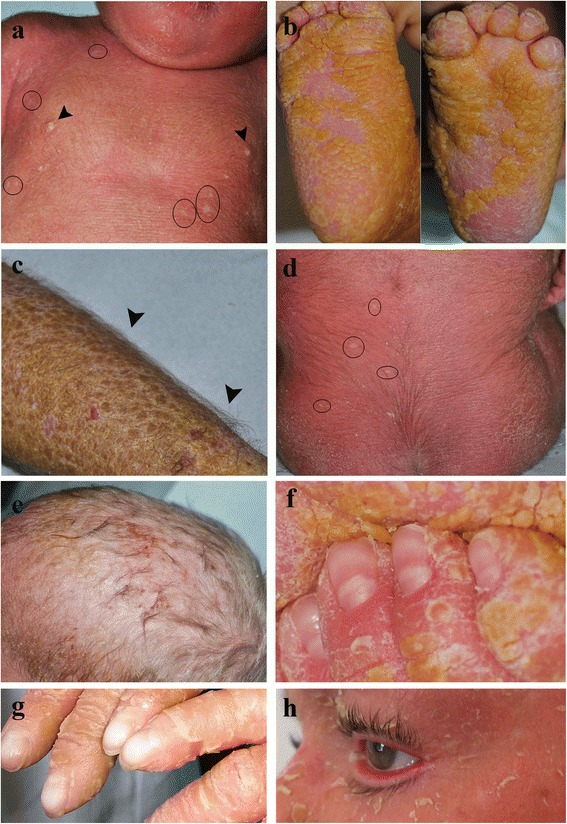
Major and minor clinical features of ichthyosis with confetti. Mammillae hypoplasia (**a**, *arrowheads*), palmoplantar hyperkeratosis (**b**), hypertrichosis of acral dorsal areas (*arrowheads*) (**c**) and back (**d**), scaling and hyperkeratosis of the scalp (**e**), large lunulae (**f**), long nail plates and unguis inflexus (**g**), severe eyelid ectropion (**h**). Clinical pictures are taken from a female at the age of 1 year (**b**, **e,** **f**), 2.5 (**c**) and 2.7 years (**a**, **d**) and a male at the age of 5 (**g**) and 8 (**h**) years. Note the healthy skin confetti-like spots (**a**, **d**: *black rings*) which became evident after hyperkeratosis shedding due to retinoid therapy

Table 1 summarizes all the clinical features, when described, in previously reported IWC patients. Ectodermal malformations, specifically ear deformities (Fig. 1e–f) and mammillae hypoplasia (Figs. 1d and 2a) are typical of IWC patients [17–19]. Ear deformities, comprising micropinna (small ears and external auditory canal) and, in a more general way, ear hypoplasia, are already evident at birth [18]. Mammillae hypoplasia has been only reported by Spoerri et al. [19]. However, it can be underestimated as Spoerri noticed that it seems visible on previous published pictures of some IWC patients [3, 12] and a reevaluation of our two patients [17] confirmed its presence (Figs. 1d and 2a).

Palmoplantar keratoderma (PPK) is associated with most IWC cases (Fig. 2b) [3, 5, 6, 10–12, 14, 17–19]. It may be characterized by an orange-red color [6, 12], increased palmar skin marks [6, 12, 18] and absent finger dermatoglyphs [14]. Disproportionately severe PPK compared with body hyperkeratosis has been reported in the IWC-II subtype [16].

Hyperpigmented, irregular-shaped macules on the erythrokeratotic skin have been reported in a few cases [3, 9]. More frequently, hyperpigmentation within healthy skin spots has been described [19].

Hypohidrosis with temperature intolerance [18] and pruritus [6, 12, 17, 19] are likely due to the ichthyosiform skin condition.

Hypertrichosis is a frequent and characteristic IWC sign [10, 11, 14, 17, 19]. It can be apparent already in early childhood, and is preferentially localized to dorsal acral areas (Fig. 2c) [17, 19], with long hair even on the back of hands and fingers [14], but can be generalized (Fig. 2d) [17]. Hypertrichosis is confined to areas of ichthyotic skin [19] and never present in confetti-like spots [14]. Scaling usually also involves the scalp (Fig. 2e) [17, 19], sometimes causing alopecia [19]. IWC patients may show reduced eyebrows and eyelashes [19] and diffuse hair loss [11]. Scalp hair heterochromia has been reported [14].

Nails may appear thickened [18], with elongated nail plates and enlarged lunulae (Fig. 2f) [19]. Subungual hyperkeratosis may induce nail curving (Fig. 2g) [3, 18, 19], the so called “unguis inflexus” [19].

Other frequent manifestations of IWC are eclabion (outward lip eversion) and ectropion (out turning of the eyelids) (Fig. 2h) [6, 11, 14, 17–19]. Additional but rarely reported signs of eye involvement are strabismus and nystagmus [19].

Finger length relative to palms may be decreased [19]. Joint contractions of the fingers, caused by marked hyperkeratosis, worsen mobility of the hands [19]. Tautness of the skin may also involve elbows, shoulders, hips and knees, thus resulting in a forced limb flexion and motor impairment with gait abnormality [16–19].

Almost all IWC individuals have small height and weight for age [1, 17, 19], but usually normal neural development. However, peripheral hyperreflexia has been detected by neurologic examination in four patients [15] and psychomotor retardation has been reported in one case [17]. Multiple non-melanoma skin cancers (NMSCs) have been described in three adult IWC patients [15].

The majority of IWC manifestations are present in the two previously reported cases of MAUIE (micropinnae, alopecia universalis, congenital ichthyosis and ectropion) syndrome (Table 1, cases 3 and 4) [7, 8]. These patients showed congenital ichthyosiform erythroderma at birth, normal-appearing skin spots, ear deformity (micropinna or external ear hypoplasia), ectropion, eclabion and nail thickening [7, 8]. At the time of examination, the two adult men also presented a complete absence of hair all over the body [7, 8]. NMSCs were additional findings in both cases [7, 8]. Although not further characterized, these patients may be considered suffering from IWC [2, 15].

Spoerri et al. [19] analysed a cohort of 6 unrelated, genetically characterized, IWC patients and suggested that clinical features of the disease may be grouped into major and minor criteria for diagnosis. Major criteria include all symptoms that the Authors identified as constantly associated with IWC in their cohort, i.e. erythroderma since birth, confetti-like spot appearance, scaling with changing severity, dorsal acral hypertrichosis, hypoplasia of mammillae and malformation of ears. Clinical features which were not always present in every patient were considered as minor criteria. Based on literature data (Table 1), palmoplantar keratoderma and ectropion appear as frequent as the major clinical findings identified by Spoerri et al. [19].

### Aetiology

IWC is a disorder of keratins. Keratins are major structural proteins of epithelial cells. They are obligate heterodimers of an acidic type I and neutral-basic type II polypeptide [21, 22]. Keratins have a characteristic expression patterns in normal human epithelial tissues according to the function and body site of each cell type. Some of them can have a very restricted tissue-specificity [23, 24]. For example, differentiating keratinocytes of the epidermis express a particular pair of type I and type II cytokeratins, keratin 10 (K10) and keratin 1 (K1) [25]. All keratins share a common structure composed of a central rod domain, through which they interact to form dimers, flanked by a N- (head) and C-terminal (tail) domain, which are important for elongation and lateral alignment of K1/K10 heterodimers [22, 25, 26]. The C-terminal “tail domain” of K1 and K10 is unusually glycine rich. In addition, the K10 tail shows extensive size polymorphism due to variable numbers and sizes of glycine loops [27]. K1/K10 heterodimers assemble to form the intermediate filaments (IF) cytoskeleton of differentiating epidermal keratinocytes [21].

IWC is due to dominant negative mutations in the K10 gene, *KRT10*, which maps to chromosome 17q21.2 and consists of 8 exons. The large majority of the 13 *KRT10* mutations identified so far represent *de novo* events. All the mutations are single nucleotide substitutions or small insertions and deletions located in exon 6, intron 6 splice sites and exon 7 (Table 1) [13, 14, 17, 19]. Of note, each mutation results in a C-terminal frameshift that converts the glycine/serine-rich K10 tail to an arginine-rich carboxy-terminal sequence [13]. As arginine-rich motifs are positively charged and encode nuclear localization sequences leading to nuclear entry, mutant K10, and its natural partner K1, accumulates within the nucleus, specifically within nucleoli, which are site of active synthesis of ribosomal RNA [13, 15].

Choate et al. [16] have recently described a new IWC subtype, named IWC-II or IWC-K1, due to a heterozygous *de novo* single base-pair insertion in the last exon of K1 gene (*KRT1*) which maps to chromosome 12q13.13. Similarly to *KRT10* gene defects, the *KRT1* mutation introduces a C-terminal frameshift, which results in the replacement of the last 22 K1 aminoacids by a novel 30-aminoacid peptide. In both IWC subtypes the frameshift peptide is implicated in the dominant negative effect that disrupts the interactions of K1/K10 tails, leading to the collapse of IF network and mislocalization of the mutant proteins to the nucleus [13, 16].

**Fig. 3:**
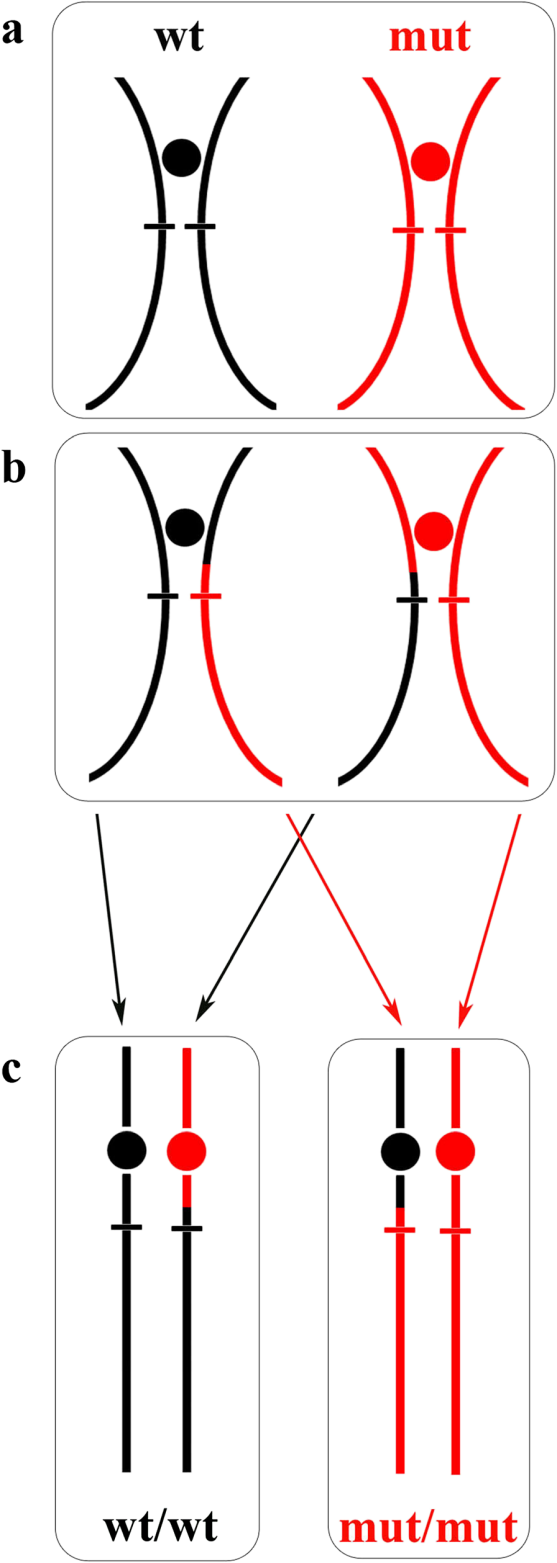
Schematic of the copy-neutral mechanism of mitotic recombination leading to revertant mosaicism in a patient affected with ichthyosis with confetti. The patient is heterozygous for a pathogenic mutation in the *KRT10* locus (17q21.2, indicated by a horizontal bar within the long arm of the chromosome). During somatic cell division, the parental affected keratinocyte bears homologous chromosomes with wild-type (wt) and mutant (mut) genotypes (**a**). Following a crossover event proximal to the *KRT10* locus both homologous chromosomes will have one chromatid carrying each genotype (**b**). Then, daughter cells receiving the same allele will be homozygous at that locus for either wild-type (revertant cell) or mutant (affected cell) genotype (**c**). A single revertant daughter cell will expand and give rise to the “confetti-like” skin spot

Of note, the *KRT10* gene mutations identified in the germline of IWC patients are not found in the DNA extracted from the “confetti-like” spots [13, 14]. Spots are not visible at birth, appear and expand over time, and have normal tissue architecture. Such observations led Choate et al. to investigate white spot-purified DNA using high-density single nucleotide polymorphism genotyping platforms [13]. In paired analysis with blood-purified genomic DNA, each DNA sample from 28 white spots taken from six independent individuals showed homozygous genotypes of a single region of chromosome 17q with copy number equals to two, indicating loss-of-heterozygosity (LOH). The delineated LOH intervals span from a proximal breakpoint to the telomere and varied among samples. However they overlapped and identified the proximal boundary of the *KRT10* locus. Thus, white spots represent “repaired” skin due to independent events of reversion of *KRT10* mutations via mitotic recombination (Fig. 3). Since white spots can be hundreds and expand over time, the recombination event is expected to occur at a high frequency and to confer selective growth advantage to revertant cell clones. A similar copy-neutral mechanism of genetic recombination was discovered in IWC-II. In this subtype the revertant tissue DNA from several revertant spots harbors overlapping LOH intervals on chromosome 12 with breakpoints proximal to the *KRT1* locus, indicating frequent somatic reversion of the *KRT1* mutation via mitotic recombination [16].

### Diagnosis

IWC diagnosis is based on dermatologic evaluation, and personal and family history. Skin manifestations, in particular the presence of verrucous hyperkeratotic plaques and associated findings such as ear and mammillae hypoplasia and hypertrichosis, should be carefully evaluated. However, IWC clinical suspicion is usually delayed until the detection of white skin spots. In addition, family history is rarely informative, as most cases are sporadic. Laboratory analyses are mandatory to confirm the diagnosis and represent the only mean to assess it before the appearance of healthy skin macules.

**Fig. 4:**
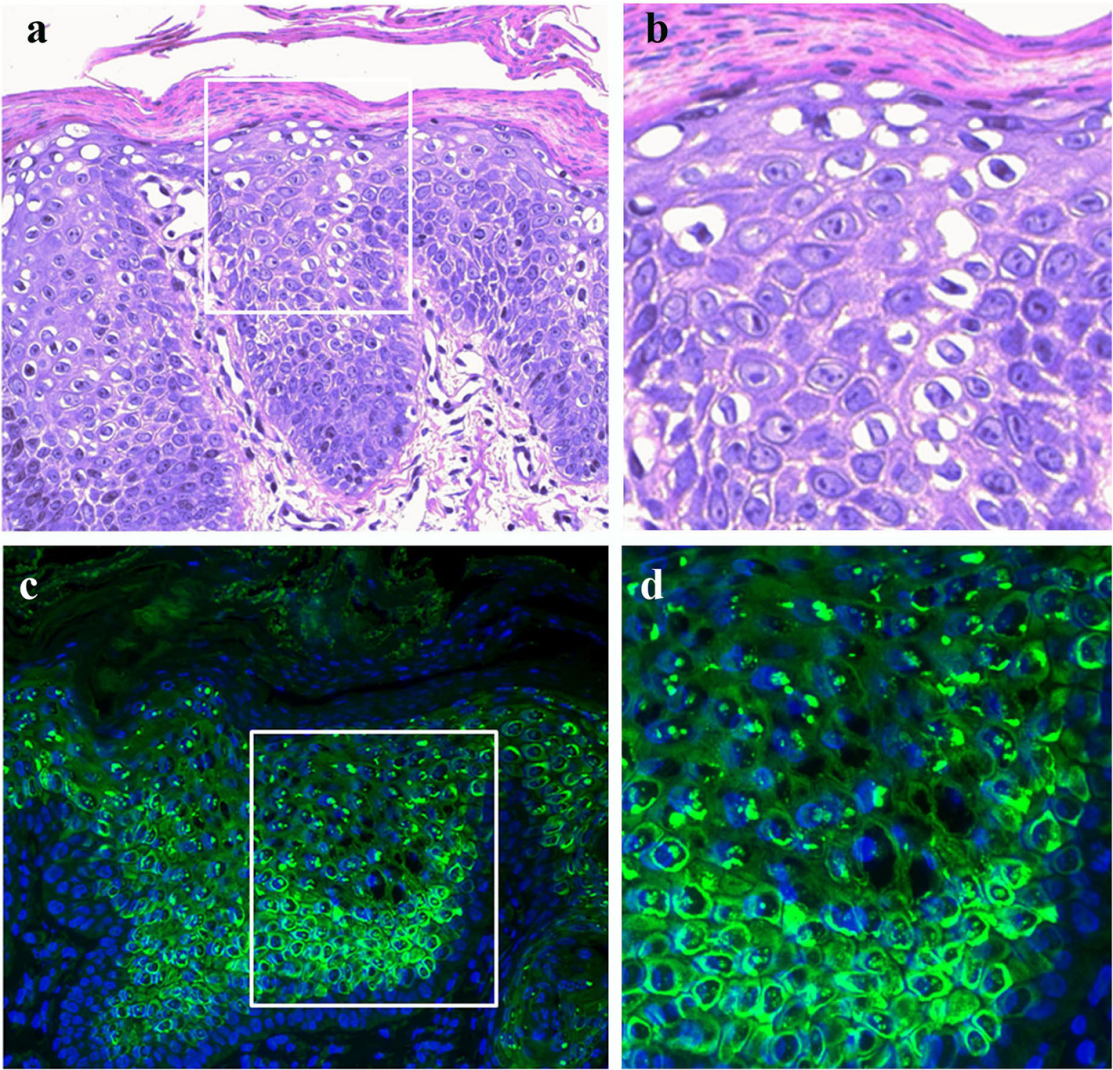
Histology and immunofluorescence findings in ichthyosis with confetti caused by *KRT10* mutation. The epidermis appears acanthotic and hyperkeratotic with parakeratosis, a reduced granular layer and cytoplasmic vacuolization in suprabasal keratinocytes (**a**). Higher magnification of the inset depicted in panel (**a**) highlighting the perinuclear vacuolization in suprabasal keratinocytes (**b**). Immunofluorescence labelling for keratin 10 (K10) shows reduced cytoplasmic staining in epidermal suprabasal cell layers, dot-like labelling of numerous nuclei in suprabasal epidermis and bright perinuclear rings (**c**). Higher magnification of the inset depicted in panel **c** showing that nuclear labeling is mainly localised to nucleoli (**d**). Haematoxylin-eosin staining (**a** and **b**), original magnification × 200 (**a**). Nuclear DAPI counterstaining (**c** and **d**), original magnification × 200 (**c**)

At first, biopsy should be taken for histological examination from affected skin. Histopathologically, the ichthyotic skin in IWC shows the following alterations of the epidermis: hyperkeratosis with focal parakeratosis (retention of cell nuclei in the stratum corneum), a reduced granular layer and pronounced perinuclear vacuolization of scattered keratinocytes in the suprabasal epidermal layers (Fig. 4a, b) [6, 11, 13, 14, 17]. The presence of binucleated keratinocytes has also been reported [3, 6]. IWC-II skin shows a thickened stratum corneum without parakeratosis, milder perinuclear vacuolization with rare binucleated cells and prominent coarse keratohyalin granules, the latter being absent in the classical IWC form [16]. On the other hand, a skin biopsy taken within a spot of normal appearing skin always reveals normal architecture. Overall, the histopathological findings of IWC, and in particular the presence of keratinocyte vacuolization, are characteristic and should prompt further diagnostic investigations. Specifically, immunopathological analysis of K10 and K1 expression in the epidermis should be performed. This is carried out by immunofluorescence labelling on formalin-fixed, paraffin-embedded skin sections with commercially available monoclonal antibodies to K10 and K1.

Immunofluorescence labelling for K10 in IWC shows a marked reduction of the cytoplasmic staining in epidermal suprabasal cell layers, collapsed filament networks visible as bright perinuclear rings in scattered keratinocytes and a dot-like labelling of numerous nuclei in suprabasal epidermis (Fig. 4c, d), while in the healthy control skin K10 is only localized to the keratinocyte cytoplasm [13, 14, 17]. Similar findings have also been described for the partner keratin, K1 [13]. Counterstaining with the nucleolar markers fibrillarin shows that K10 and also K1 localize to the nucleolus [13]. Immunofluorescence labelling for K1 in the IWC-II subtype reveals perinuclear rings in affected skin [16]. The nuclear staining for K10 has been described only in IWC epidermis and can be considered a diagnostic hallmark. In addition, K10 immunolabelling performed on archival skin biopsies can allow retrospective diagnosis [17].

At the ultrastructural level, the most significant findings in IWC are the presence of binuclear keratinocytes and cup- or bowl-like perinuclear masses of granular material in the vacuolized superficial keratinocytes [3, 6, 9, 12]. A closer examination demonstrates that this material is composed by thin interlacing filaments [6, 9, 12]. Additional electron microscopy abnormalities include marked reduction in the total number of keratin tonofilaments in cytoplasm, poorly formed desmosomes and sparse and tapered bundles of keratin filaments attached to desmosomes [13].

**Fig. 5:**
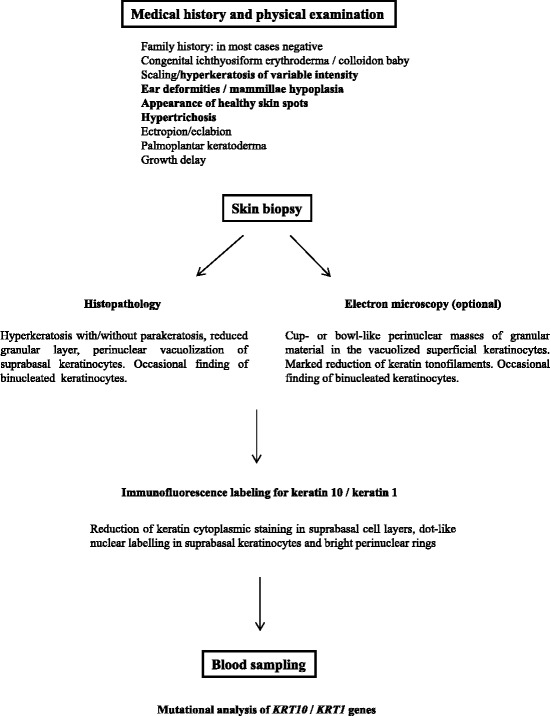
Proposed diagnostic algorithm for ichthyosis with confetti. Medical history and physical examination raise diagnosis suspicion. Histological examination and keratin immunolocalization show specific findings, such as suprabasal keratinocyte vacuolization and nuclear keratin staining, respectively. Electron microscopy may further support the diagnosis. Finally, mutational analysis of *KRT10/KRT1* genes represents the gold standard to confirm the diagnosis. Findings relevant to differential diagnosis are in *bold*

Altogether, histopathological and immunofluorescence findings of IWC are pathognomonic. Electron microscopy may further support the diagnosis. Finally, mutational analysis of *KRT10* and *KRT1* genes is at present the gold standard to confirm IWC diagnosis and provides a firm basis for genetic counseling of affected individuals and families. Figure 5 summarizes the proposed diagnostic algorithm for IWC.

### Differential diagnosis

IWC at its onset is usually confused with the erythrodermic form of autosomal recessive congenital ichthyosis (ARCI), congenital ichthyosiform erythroderma (CIE) [4, 28]. The following clinical criteria should be used for the differential diagnosis of IWC: i. the evolution over time of skin manifestations, in particular a progressive worsening of the hyperkeratosis may be suggestive of IWC; ii. the type of scaling and hyperkeratosis, as severe, adherent hyperkeratotic plaques are not usually seen in CIE; iii. the presence of additional dermatologic features, in particular ear and mammillae hypoplasia, and hypertrichosis; iv. the family history which may point toward a particular mode of inheritance and, above all, v. the appearance of confetti-like spots which drives the correct diagnosis. Histopathological and immunopathological features also allow to differentiate IWC from CIE. Indeed, keratinocyte vacuolization and K10 reduced cytoplasmic labeling and nuclear staining are not found in CIE. Table 2 summarizes differences between CIE and IWC [4, 29, 30]. IWC can also be differentiated from syndromic ichthyoses, in particular Netherton syndrome [31] which manifests at birth with ichthyosiform erythroderma, but also presents specific hair alterations (i.e. trichorrhexis invaginata). The absence of overt skin blistering in infancy allows to distinguish IWC from other keratinopathic ichthyoses [4].

**Table 2:** Differential diagnosis between congenital ichthyosiform erythroderma (CIE) and ichthyosis with confetti (IWC) / congenital reticular ichthyosiform erythroderma (CRIE)

	CIE	IWC (CRIE)
Classification	Non-syndromic autosomal recessive congenital ichthyosis (ARCI)	Non-syndromic congenital ichthyosis
Mode of inheritance	Autosomal recessive	Autosomal dominant
Mutated genes	^a^ *ABCA12, ALOXE3, ALOX12B, CERS, CYP4F22, NIPAL4, PNPLA1, TGM1*	*KRT10*, *KRT1*
Gene function	Epidermal lipoxygenase/hepoxilin metabolism, ceramide synthesis, cornified envelope precursor cross-linking	Intermediate filament assembly
Clinical findings		
Onset	At birth	At birth
Initial clinical presentation	CIE or, less frequently, collodion baby	CIE or collodion baby
Disease course	Ranging from mild to severe	During childhood numerous spots of normal skin manifest and are the hallmark of the disease. A later age for normal skin spot appearance characterizes IWC caused by *KRT1* mutation
Distribution of scaling	Generalized, focally pronounced scaling possible	Verrucous adherent hyperkeratosis, more evident on limbs, possible. Later reticular ichthyosiform pattern
Scaling type/color	Fine/white or gray	Fine to coarse, yellow-brown
Erythema	Variable, often pronounced	Pronounced
Palmoplantar involvement	Mild to pronounced	Mild to pronounced
Hypohidrosis	Moderate to severe	Reported in some cases
Scalp abnormalities	Scarring alopecia possible	Scaling alopecia possible, hair loss and alopecia universalis reported
Other skin findings	Rarely ectropion	Hypertrichosis, ectropion, eclabion
Associated findings	Failure to thrive, short stature (if severe)	Ear deformities, mammillae hypoplasia, growth failure
Risk of death	Present during neonatal period	Elevated during neonatal period
Histopathology	Hyperkeratosis with occasional parakeratosis, normal or thickened granular layer, pronounced acanthosis	Hyperkeratosis with/without (*KRT10*/*KRT1*-subtypes) parakeratosis, reduced or absent granular layer, pronounced perinuclear vacuolisation of suprabasal keratinocytes; coarse keratohyalin granules in *KRT1-*subtype.
Immunopathology	No specific findings	Reduction of keratin cytoplasmic staining in epidermal suprabasal cell layers, dot-like labelling of numerous nuclei in suprabasal epidermis, bright perinuclear rings in scattered keratinocytes

^a^ *ABCA12* ATP-binding cassette subfamily A12; *ALOX* arachidonate lipoxygenase; *CERS3* ceramide synthase 3; *CYP4F22* cytochrome P450 4 F22; *NIPAL4* NIPA-like domain containing 4; *PNPLA1* patatin-like phospholipase domain-containing protein 1; *TGM1* transglutaminase-1; *KRT10*/*KRT1* keratin 10/1 [4, 28, 29]

### Genetic counselling

Genetic counselling in IWC is usually required by the dermatologist who has in charge the affected patient/family. It is aimed to: i. confirm the diagnosis, ii. ascertain disease recurrence risk in future pregnancies, and iii. plan prenatal diagnosis. Histopathological and immunopathological analyses should be considered as first steps in the diagnostic pathway as they permit to put in evidence the peculiar tissue architecture and keratin nuclear staining of affected skin, thus allowing early diagnosis before revertant skin spots become evident [17]. Then, molecular analysis of *KRT10* and, if IWC-II subtype is suspected, of *KRT1* gene is aimed at identifying the heterozygous frameshift mutation in the hotspot genomic region coding for the C-terminal tail of keratins 10 and 1. Mutation identification provides confirmation of the disease subtype and allows to support the autosomal dominant mode of inheritance, which has a 50 % recurrence risk for subsequent pregnancies of affected individuals. Knowledge of the mutation also provides a tool to monitor recurrence risk by prenatal testing. However, IWC often manifests as a sporadic case, making difficult the diagnosis in infancy when “confetti like” spots are not yet evident and, thus, CIE is usually suspected. The latter is classically inherited as an autosomal recessive trait, which bears a 25 % recurrence risk for subsequent pregnancies, while sporadic IWC has a lower risk of recurrence as it results from *de novo* event(s) during gametogenesis or at conception. Therefore, molecular analysis is the only way to properly counsel the couples in sporadic IWC. Molecular confirmation after diagnosis by immunofluorescence also provides a prenatal diagnostic tool in selected pregnancies to exclude gonadal mosaicism.

### Management including treatment

Due to the rarity of the disease, there are no controlled studies on IWC management. In general, care of the disease follows the rules for treatment of ARCI patients.

Like for other congenital ichthyosis forms, the management of IWC in the neonate, presenting either with ichthyosiform erythroderma or colloidon baby, requires admission to a neonatal intensive care unit and a multidisciplinary approach, with nursing staff, neonatologists, dermatologists and other specialists [32]. In particular, the skin barrier function of the newborn is greatly compromised and ongoing water loss can lead to dehydration and electrolyte imbalance [33]. Moreover defective barrier allows bacteria and yeast colonization, increasing risks of infection and sepsis [17]. Thus, neonate care includes providing a temperature controlled environment via a humidified incubator, and frequent application of lubricants such as petrolatum-based products under sterile conditions [32, 33]. Urinary output, electrolytes and weight should be monitored and managed with intravenous hydration, electrolyte repletion and additional nutritional input when necessary. Close monitoring and prompt systemic treatment with antibiotics for infection is essential.

Therapy of infants, children and adults affected with ichthyosis, including IWC, is not curative but rather aimed at symptom relieving. Daily bathing with water and sodium bicarbonate alkalinizes the epidermis and is helpful for many patients, especially for mechanical scale removal. If sodium bicarbonate is not tolerated, rice starch can be used and is preferred in the first year of life. During bathing, gentle mechanical keratolysis can be obtained using sponges or microfiber cloths. Bland emollients such as petrolatum-based products should be applied several times a day, especially after bathing, to prevent drying. Starting from the second year of life, keratolysis may be obtained through alpha-hydroxy (e.g. lactic and glycolic) acids and urea or combination of these ingredients. Alpha-hydroxy acids-based lotions reduce corneocyte adhesion and skin thickness and relieve itching. Urea creams decrease dryness and scaling and improve skin permeability barrier function by regulating epidermal gene activity [34]. Of note, topical urea and alpha-hydroxy acids can also cause skin irritation and should be applied with caution and only on stubborn areas in the first three years of life. Moreover, alpha-hydroxy acids and urea creams may cause systemic absorption with metabolic acidosis or elevating plasma urea levels, respectively, when applied over large body surfaces in infants and toddlers [35, 36]. The use of a topical retinoid, tazarotene cream, has been reported in a single IWC patient, specifically on skin areas presenting marked hyperkeratosis and around eyes to prevent ectropion [18]. Although topical tazarotene is increasingly used in ichthyosis patients [37, 38], it can be irritating [37, 39] and thus not tolerated in erythrodermic forms of the disease. Its use in IWC should be therefore cautiously considered for selected skin areas, such as hyperkeratotic ones or eyelids, and tolerance evaluated in each patient. Despite low systemic absorption [40], rules for pregnancy avoidance during systemic retinoid treatments should also be applied to tazarotene topical therapy.

Due to disease severity, oral retinoids, at first etretinate [1, 3, 6] and then acitretin [11, 17], have been used in most IWC patients with positive results, including extension of the areas of normal confetti-like skin [1, 6, 17]. Systemic retinoids decrease cell proliferation, thus thinning the stratum corneum, normalize keratinocyte differentiation, facilitate desquamation through downregulation of desmosomal proteins, and have anti-inflammatory properties [41, 42]. Of note, they have been also shown to downregulate K10 expression [41, 43]. However, the lowest possible dose of a systemic retinoid producing desired clinical outcomes needs to be titrated. Few IWC patients require more than 0.5 mg/kg of acitretin once a day [11, 17]. Importantly, in women of childbearing age pregnancy must be excluded by negative pregnancy test (serum levels of human chorionic gonadotropin) within 2 weeks prior to therapy and effective contraception has to be initiated 4 weeks before, during and for 3 years after retinoid therapy [44].

Acute adverse effects of systemic retinoids include skin irritation, fragility and tenderness, mucocutanous toxicities (in particular cheilitis, epistaxis, eye irritation), hair loss, and laboratory abnormalities in blood cell counts, transaminases and serum lipids [44]. Complete blood count, liver function, cholesterol and lipid levels must be monitored.

Possible long-term retinoid complications include premature epiphyseal closure, hyperostosis and tendon calcifications [42, 44]. Although not reported to date in IWC, they represent an indication to limit the duration of retinoid therapy or to periodically interrupt their administration, e.g. during summertime. Baseline radiographs should also be obtained before programming long-term retinoid therapy. Frequency of X-ray follow-ups, complemented if required with bone density scan, will depend on baseline findings and on clinical manifestations and symptoms. In addition, it has been hypothesized that retinoids might interfere with vitamin D metabolism, as their vitamin A-like biologic activity may hinder vitamin D action [45]. Of note, vitamin D deficiency has been described in most types of ichthyoses, in particular severe forms, and attributed to different factors, such as poor sunlight penetration due to hyperkeratotic skin and limited sun exposure for cosmetic reasons or sun-induced pain and pruritus [46]. Therefore, it is currently recommended to screen all congenital ichthyoses for calcium, phosphorus and vitamin D3 levels and to provide supplementation if required, in order to treat vitamin D3 deficiency and prevent possible complications, such as osteoporosis and rickets. This recommendation should apply also to IWC, although no data on ions and vitamin D3 levels in these patients have been reported to date.

For infection control in IWC, topical antiseptics and antimicrobials must be combined with appropriate systemic therapy. Among other IWC complications, severe ectropion should be managed by an ophthalmologist to avoid desiccation, e.g. through regular application of liquid tears and eye lubricants, and plastic surgery may be required. Routine otolaryngologist visits are required to remove desquamated skin from ear canals. Contractures and gait abnormalities should be evaluated by a combined team of orthopedists, neurologists and plastic surgeons and physical therapy should be instituted for patients with these findings. Psychological support may be of benefit to both patients and their families. Regular examination for skin cancer risk is recommended in adulthood.

### Prognosis

IWC, as most other heritable ichthyoses, is a condition that requires significant attention in the neonatal period. Although at present there is no curative therapy for all forms of ichthyoses, including IWC, treatments have improved considerably over the years. Topical medications can reduce scaling and, thus, patient quality of life. In addition, retinoid therapies decrease scaling and hyperkeratosis, and may help in preventing and treating ectropion. Nevertheless, treatments remain symptomatic and topical ones are time-consuming, challenging patient and caregiver compliance. Therefore, personalized multidisciplinary care plans should be set up and regularly up-dated by combined dermatologist and patient efforts. In this context, the support of patient organizations (see Additional file 1: Table S1) will help affected individuals and their families to better cope with the disease.

### Unresolved questions

There have been recently major advances in our understanding of the genetic basis of IWC, with the identification of *KRT10* and *KRT1* mutations. However, several questions remain unanswered. First, although IWC is considered a nonsyndromic ichthyosis, it is also true that a spectrum of ectodermal malformations and neurological findings leading to different degrees of disability have been described in affected individuals without any apparent genotype-phenotype correlation. Indeed, among 40 previously reported IWC patients, 7 showed both ear deformities and mammillae hypoplasia [17, 19], whereas 6 presented ear deformities [7, 8, 18] or mammillae hypoplasia [3, 12, 19] alone (Table 1). Moreover, 4 out of 40 IWC cases were diagnosed with peripheral hyperreflexia [15] (Table 1). This led some Authors to suggest a reclassification of IWC as a syndromic ichthyosis [19].

Mutations affecting K10/K1 heterodimers trigger keratinocyte hyperproliferation [24], which explains acanthosis and hyperkeratosis in IWC patients. Although K10/K1 are not expressed in nail bed, they are detectable in all other regions of the nail unit, including eponychium, hyponychium and the apical matrix [47, 48]. Therefore *KRT10/KRT1* mutations may also cause nail dystrophies in IWC patients. In contrast, K10/K1 are absent from the hair follicle, except for the infundibulum [47], thus raising the question of pathogenetic mechanisms underlying hypertrichosis in IWC. Hypertrichosis is limited to areas of ichthyotic skin and Spoerri et al. hypothesized that it may be due to inflammation and hyperemia, as it happens in postcast hypertrichosis [19].

Another open issue is the timing of mitotic recombination leading to the generation of revertant cells. Spots become evident as early as 2.5 years of age [17] and can reach a diameter of 4 cm in adulthood [13]. Since a white spot represents the expansion of a single homozygotic revertant daughter cell in the absence of phenotypic evidence of homozygotic mutant daughter cell [13], the recombination event should involve the epidermal stem cell unit, the specification of which is thought to occur during embryogenesis [49]. This leads to think that generation of revertant cells might begin already in fetal skin. Moreover, as mutant K10 is thought to be involved in triggering the recombination event, *KRT10* should be transcriptionally active in the epidermal stem cell. However, evidence of *KRT10* expression in epidermal stem cells is still quite limited [50].

A further intriguing question concerns the extremely high rate of mitotic recombination. As different types of keratin mutations lead to keratinization diseases without revertant mosaicism, this implicates the C-terminal frameshift peptide of mutant K10 and K1, and its nuclear localization in the elevated rate of reversion in IWC [15]. Interestingly, revertant spots associated with IWC-II are lesser in number and smaller in size compared to classic IWC and harbor clinically apparent affected skin islands [16]. Moreover, spot-derived keratinocytes of IWC-II give rise to mixed cultures of revertant and mutant cells, thus revertant cells seem to have a lesser growth selective advantage [16]. Finally, nuclear K1 staining is clearly evident in epithelial cells transfected with mutant K1, but not in IWC-II patient epidermis [16]. The explanation for these observations might be dual: i. the sequence of the mutant C-terminal frame-shifted K1 peptide harbors very few positive-charged residues compared to mutant K10 and ii. only the K10 protein has been linked to cell cycle control [24, 51, 52], suggesting that mutant K10 might gain and/or loss an activity that interferes with this process. However, the relationship between IWC *KRT10/KRT1* mutations and the frequency of mitotic recombination events leading to revertant cells remains to be determined.

Adult individuals with IWC may also have a higher risk to develop NMSCs [7, 8, 15]. Although such risk should be carefully re-evaluated by long term patient follow-up, tumour susceptibility in IWC may have a genetic rationale related to the mechanism of somatic mosaicism. Indeed, LOH occurring on chromosome 17q not only involves the *KRT10* locus but can also reduce to homozygosity mutations and/or variants in epithelial cancer susceptibility loci distal to the *KRT10* allele. For example, within chromosomal band 17q21.31 several of these loci have been mapped, including the *BRCA1* tumor suppressor [53, 54]. Homozygotic revertant cells might have a selective growth advantage also as a consequence of LOH events in tumor suppressor loci. However, available data on *BRCA1* mutations and non-melanoma skin cancer show non-significant association [54]. In addition, it is not known whether squamous cell carcinomas reported in IWC individuals developed from revertant or erythrokeratotic skin. Finally, NMSCs have been reported also in other ichthyoses, in particular erythrodermic ones, which do not present revertant mosaicism [55, 56].

### Future perspectives

Revertant skin spots could be exploited in future as a cell source for a “natural gene therapy” approach in IWC. The feasibility of *ex-vivo* gene therapy for genetic skin disorders has been demonstrated by the successful engraftment of *LAMB3* cDNA retrovirally-corrected epidermal sheets and maintenance of a functional epidermis in a 36-year old male with non-Herlitz junctional EB [57, 58]. However, concerns remain regarding the safety of this approach as there is an oncogenic potential related to random insertion of retroviral vectors [59]. By contrast, the natural occurrence of revertant mosaicism creates a unique opportunity for therapy in patients, because the presence of reverted cells circumvents the need for viral vectors. Persistent ulcers in a patient with non-Herlitz junctional EB, caused by mutations in the *LAMB3* gene, have been recently treated by transplantation of punch biopsy specimens taken from one of his revertant patches with complete re-epithelialization and restoration of a healthy phenotype and genotype in the grafted areas [60]. Stable reversion of the EB phenotype by transplantation of revertant skin in this patient may be a pioneering work for the development of the “revertant cell therapy” for other genetic diseases with somatic revertant mosaicism, such as IWC. To date, the only attempt to utilize revertant cell therapy in a clinical setting was performed in an individual with non-Herlitz junctional EB associated with mutations in *COL17A1* who presented revertant skin patches [61]. In this case, revertant keratinocytes were isolated and expanded into epidermal sheets that were subsequently grafted back onto the patient. However fewer than 3 % of the cells remained corrected in the graft and there was no clinically relevant outcome.

The persistence of revertant clones in patients with revertant mosaicism indicates that reversion events occur in epidermal stem cells and that naturally-corrected stem cell clones may be under strong positive selection [13, 62]. Therefore, efficacy and long-term persistence of the regenerated epidermis after autotransplantation requires epidermal stem cell maintenance in cultured sheets and their grafting onto the patients [63]. We hypothesize that, with optimized culture conditions, the “revertant cell therapy” might be applied in future to IWC. Nevertheless, in this scenario, the possibility of homozygosity for mutations in tumor suppressors should be carefully considered together with the potential therapeutic advantage of reversion.

## Conclusions

IWC is a very rare genodermatosis and represents the most impressive example of revertant somatic mosaicism. The IWC phenotype is characterized by a wide spectrum of clinical features, in addition to erythrodermic ichthyosis. The recent discoveries in IWC genetics have led to a better understanding of disease aetiology and made available molecular diagnostic tools. Disease care requires a multidisciplinary approach, but remains symptomatic. However, based on current scientific and technological progresses, new therapeutic strategies potentially able to cure skin manifestations might be developed in future.

### Consent

Written informed consent for patient reexamination for the present review and for patient data and image publication was obtained from patients’ parents.

## Additional file

Additional file 1: Table S1. Worldwide ichthyosis foundations and patient organizations. (DOC 31 kb)

## Abbreviations

IWC: Ichthyosis with confetti
CRIE: Congenital reticular ichthyosiform erythroderma
CIE: Congenital ichthyosiform erythroderma
MAUIE: Micropinnae, alopecia universalis, congenital ichthyosis and ectropion
PPK: Palmoplantar keratoderma
NMSC: Non-melanoma skin cancer
K/*KRT*: Keratin (protein/*gene*)
IF: Intermediate filaments
LOH: Loss of heterozygosity
ARCI: Autosomal recessive congenital ichthyosis
