# Supplementary material for: Ichthyosis with confetti: clinics, molecular genetics and management
Source: Orphanet J Rare Dis. 2015 Sep 17;10:115. doi: 10.1186/s13023-015-0336-4 (PMC4573700; doi:10.1186/s13023-015-0336-4)
Supplement: Additional file 1: Table S1. — Worldwide ichthyosis foundations and patient organizations. (DOC 31 kb) [file 13023_2015_336_MOESM1_ESM.doc]

**Table S1.** Worldwide ichthyosis foundations and patient organizations

FIRST-Foundation for Ichthyosis & Related Skin Types [http://www.firstskinfoundation.org](http://www.firstskinfoundation.org/)

(formerly NIF-National Ichthyosis Foundation) (USA**)**

ENI-European Network For Ichthyosis http://www.ichthyose.eu

- ASIC-Spanish Association of Ichthyosis (Spain) http://www.ictiosis.org
- ASPORI-Associaçao Portuguesa de Portadores de Ictiose (Portugal) http://aspori.blogspot.fi
- AIF-Association Ichtyose France (France) http://ichtyose.fr
- Ichthyosis in Ireland (Ireland) http://www.facebook.com/pages/Ichthyosis-in-Ireland 107619475940859
- Ichthyosis Stichting België VZW (Belgium) http://www.devidts.com/ichthyosis
- ISG-Ichthyosis Support Group (United Kingdom) http://www.ichthyosis.org.uk
- Iktyoseforeningen I Norge (Norway) http://iktyose.no
- Iktyosföreningen (Sweden) [http://www.iktyos.se](http://www.iktyos.se/)
- Iktyosisforeningen I Danmark (Denmark) http://www.iktyosis.dk
- PSI-Polish Ichthyosis Society (Poland) http://www.ichtiozis.fora.pl
- Selbsthilfe Ichthyose E.V. (Germany) http:// www.ichthyose.de
- Suomen Iktyoosiyhdistys (Finland) http://www.iholiitto.fi
- UNITI-Unione Italiana Ittiosi (Italy) http://www.ittiosi.it
- Vereinigung Ichthyose Schweiz (Switzerland) http://www.ichthyose.ch/de

Chinese Ichthyosis Association http://www.ylbhome.com

Japanese Ichthyosis Association http://www.gyorinsen.com

Swedish Site (All for Parents) http://www.alltforforaldrar.se

ANIPS-French National Association of the Ichthyoses and Pathological Dry Skin (France) http://www.anips.net

Friends of Ichthyosis (United Kingdom) http://www.friends-of-ichthyosis.webs.com

New Zealand Support Group - Rachel Davidson (New Zealand) ra.davidson@xtra.co.nz

SHHIRT - Samuel Hardgrave Harlequin Ichthyosis Research Trust (United Kingdom) http://www.shhirt.org.uk

Association ATHINA Ichtyose Monaco (Monaco) [http://www.aaimonaco.org](http://www.aaimonaco.org/adresse-utile.php)
